# Utility of the Aintree Intubation Catheter in fiberoptic tracheal intubation through the three types of intubating supraglottic airways: a manikin simulation study

**DOI:** 10.1007/s00540-013-1724-3

**Published:** 2013-10-17

**Authors:** Ryusuke Ueki, Nobuyasu Komasawa, Kenta Nishimoto, Takashi Sugi, Munetaka Hirose, Yoshiroh Kaminoh

**Affiliations:** 1 Department of Anesthesiology, Hyogo College of Medicine, Mukogawa-cho 1-1, Nishinomiya, Hyogo 663-8501 Japan; 2 Department of Surgical center, Hyogo College of Medicine, Mukogawa-cho 1-1, Nishinomiya, Hyogo 663-8501 Japan; 3 Department of Anesthesiology, Osaka Medical College, Daigaku-machi 2-7, Takatsuki, Osaka 569-8686 Japan; 4 Department of Anesthesiology, Yamashiro Public Hospital, Kizuekimae 1-27, Kizugawa, Kyoto 619-0214 Japan

**Keywords:** Aintree Intubation Catheter (AIC), Supraglottic airway devices for tracheal intubation (ISGA), Fiberoptic intubation

## Abstract

**Purpose:**

The purpose of this study was to examine the utility of the Aintree Intubation Catheter (AIC) with three types of supraglottic airway devices for tracheal intubation (ISGAs) using a manikin.

**Methods:**

Participants were 21 anesthesiologists with more than 2 years of experience in clinical anesthesia. Three types of ISGAs were passed through the glottis: Fastrack-Single Use (FSU; size 4), air-Q (size 3.5), and i-gel (size 4). Participants attempted fiberoptic tracheal intubation with the ISGAs in random order. Success rate of tracheal intubation, intubation time, and collision with the glottis were recorded. Participants also evaluated the subjective difficulty of the entire intubation process and passing the tracheal tube through the glottis using a Visual Analogue Scale.

**Results:**

The FSU required a significantly longer time for intubation compared with the other two ISGAs (*p* < 0.05). AIC use did not significantly improve the success rate of intubation or shorten intubation times for any of the ISGAs. However, there were significantly more collisions with the glottis without AIC use for the FSU and air-Q compared to when they were used with the AIC (FSU, *p* = 0.015; air-Q, *p* = 0.025).

**Conclusion:**

Among the ISGAs tested, intubation took longer with the FSU, and the FSU had a higher failure rate than the other ISGAs. AIC significantly decreased the number of collisions with the FSU and air-Q. These findings suggest that the AIC is effective in reducing collisions with the tracheal tube and thus will reduce the risk of mechanical injury to the airway.

## Introduction

Various supraglottic airway devices (SGAs) have been developed to secure the airway [1–3]. Clinical [4–8] and manikin [9–11] studies have demonstrated that SGAs adequately and effectively secure both normal and difficult airways. However, each SGA has its own limitations regarding adaptability, and fiberoptic intubation remains one of the most important methods for securing difficult airways [12, 13]. The combined use of SGAs and a fiberscope is considered an effective way to manage difficult airways [14–16]. Although there are few weak points of fiberoptic intubation, a remaining problem is that insertion of tracheal tube may be prevented by structures around the vocal cords such as the arytenoids [17].

**Fig. 1:**
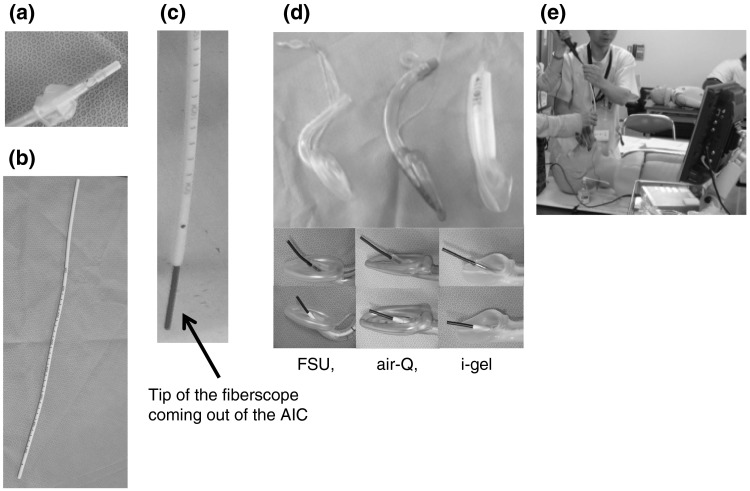
**a** Aintree Intubation Catheter (AIC) through a Portex (7.0-mm) tracheal tube. **b** AIC: external diameter, 19 Fr.; internal diameter, 4.5 mm; working length, 56 cm. **c** Tip of fiberscope coming out of the AIC. Fiberscope has external diameter of 3.4 mm and working length of 60 cm. **d** Three ISGAs: Fastrack-Single Use (FSU; size 4), air-Q (size 3.5), and i-gel (size 4.0). Fiberscope through the tracheal tube and each ISGA (*upper* without AIC, *lower* with AIC). **e** Fiberoptic intubation with the AIC

The Aintree Intubation Catheter (AIC) is an instrument used to guide the tracheal tube with the assistance of a fiberscope (Fig. 1). Numerous studies have reported that the AIC is useful for tracheal intubation through the conduit of an SGA [18–20]. Moreover, we consider that the AIC may help the passing of tracheal tube through the glottis, because the AIC might shift the fiberscope to the center of the larynx and fill in the gap between the fiberscope and tracheal tube.

In the present study, we aimed to assess the utility of the AIC such as during fiberoptic intubation of a manikin using three SGAs for tracheal intubation (ISGAs).

## Materials and methods

The Institutional Review Board of Hyogo College of Medicine approved this study (No. 1332), and written informed consent was obtained from each participant. A total of 21 medical doctors, each with more than 2 years of clinical experience in anesthesia, were invited to participate. Each participant attempted fiberoptic intubation six times using three ISGAs, with or without AIC use, in random order by lottery: LMA Fastrack-Single Use (FSU; size 4; The Laryngeal Mask Company, San Diego, CA, USA), air-Q (size 3.5; Mercury Medical, Clearwater, FL, USA), and i-gel (size 4.0; Intersurgical, Berkshire, UK) (Fig. 1d, e).

The time limit for tracheal intubation was set at 3 min, and giving up during an intubation attempt was considered a failed attempt. The following were used in the trials: the Airway Management Trainer (Laerdal, Norway) manikin for a model of an adult male by referring to the product explanation and previous studies [21, 22], a 7.0-mm-internal-diameter Portex tracheal tube (Smiths Medical, London, UK), a bronchofiberscope with an external diameter of 3.4 mm and working length of 60 cm (Pentax, Tokyo, Japan), and a 19 Fr. AIC with external diameter of 6.3 mm, internal diameter of 4.5 mm, and working length of 56 cm. Photographs of the tracheal tube, AIC, and fiberscope are shown in Fig. 1. A video system (Machida Endoscope, Tokyo, Japan) was used as a monitor for the fiberscope.

Olive oil (Yoshida Pharmaceutical, Saitama, Japan) was spread on the surface of the fiberscope and AIC as a lubricant, and 8 % lidocaine (AstraZeneca, London, UK) was only used to lubricate the inside of the tracheal tube. Before measurements, the fiberscope was set in the tracheal tube with or without the AIC, and the tip of the fiberscope was placed 16 cm (FSU) or 19 cm (air-Q and i-gel) from the entrance of each SGA.

An experienced anesthesia specialist judged the success of the procedure and measured the intubation time for each trial. The start point of intubation was when the fiberscope began to advance, and the end point was when tracheal intubation was confirmed by the fiberscope. The number of collisions of the tracheal tube with the glottis was also recorded. Participants evaluated the difficulty of the entire intubation procedure and passage through the glottis using a Visual Analog Scale (VAS).

Sample size was calculated based on a preliminary study that evaluated the time required for eight participants to complete intubation. The longest and second longest mean (SD) times required to confirm successful tracheal intubation were 49.4 (18.1) s and 29.1 (12.6) s. To detect a 33 % difference in intubation time with a power of 0.8, we estimated that 13 operators would be adequate for each device.

### Statistical analysis

Data were expressed as mean ± standard deviation and analyzed with two-way repeated analysis of variance (ANOVA), Tukey’s multiple comparison test for intubation time, the chi-square test for procedure success rate, and the Kruskal–Wallis test for the number of collisions of the tracheal tube with the glottis. *p* < 0.05 was considered significant.

## Results

### Intubation success rate (Table 1)

Although there were more successful intubations with the FSU-AIC (19/21) than with the FSU alone (16/21), the difference was not significant (*p* = 0.21). There were no failures with the other ISGAs, suggesting that intubation with the FSU was associated with more failures than with the other two devices (*p* = 0.017).

**Table 1:** Number of successful intubations/all intubations

	Without AIC	With AIC
FSU	16/21*	19/21
air-Q	21/21*	21/21
i-gel	21/21*	21/21

*FSU* Fastrack-Single-Use size 4, *airQ* air-Q size 3.5, *i-gel* i-gel size 4, *AIC* Aintree Intubation Catheter

^*^ *p* = 0.017 (FSU vs. air-Q, i-gel)

### Intubation time

**Fig. 2:**
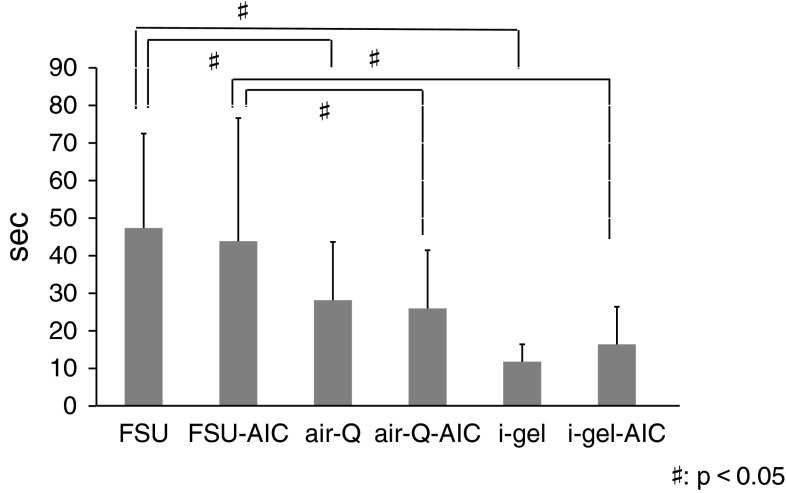
Comparison of mean intubation time (*n* = 21). There were 5 failed attempts with the FSU and 2 failed attempts with the FSU-AIC

Intubation times for the ISGAs are shown in Fig. 2. Intubation time with the FSU, both with and without AIC use, was the longest compared with the other two ISGAs (*p* < 0.05). However, AIC use did not significantly shorten intubation times for any of the ISGAs.

### Collision with the glottis

**Fig. 3:**
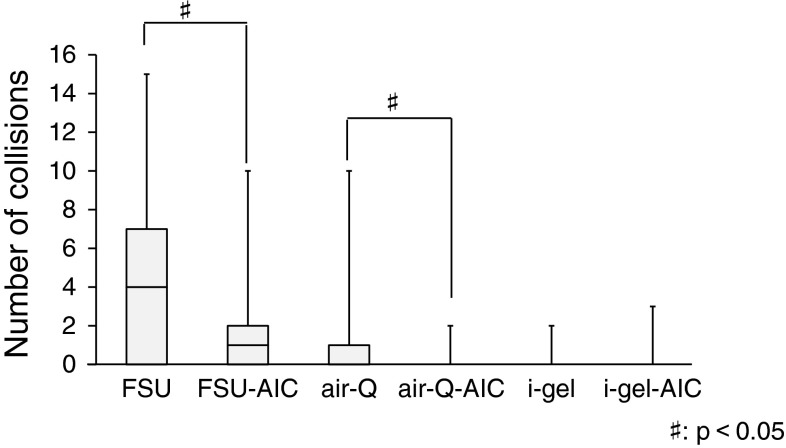
Number of collisions with glottis (*n* = 21): 5 failed attempts with the FSU and 2 failed attempts with the FSU-AIC

Figure 3 shows a box plot graph of the number of collisions with the glottis for each ISGA, with or without AIC use. FSU and FSU-AIC had more collisions compared with the other two ISGAs (*p* < 0.001). Moreover, there were more collisions without AIC use than with AIC use with the FSU and air-Q (FSU, *p* = 0.015; air-Q, *p* = 0.025).

### Difficulty of intubation and passing through the glottis

**Fig. 4:**
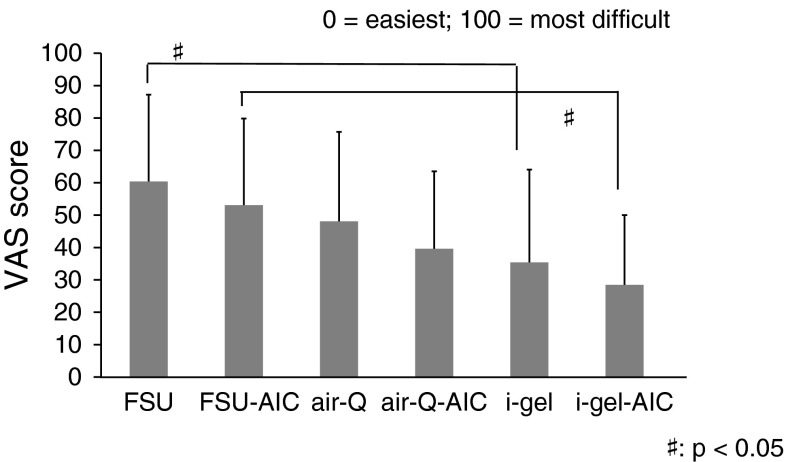
Difficulty of the entire procedure (*n* = 21)

Figure 4 shows VAS scores for the difficulty of the entire intubation procedure. “0” was defined as easiest and “100” as the most difficult. The FSU had a significantly higher score than the i-gel both with and without AIC use (*p* < 0.05).

**Fig. 5:**
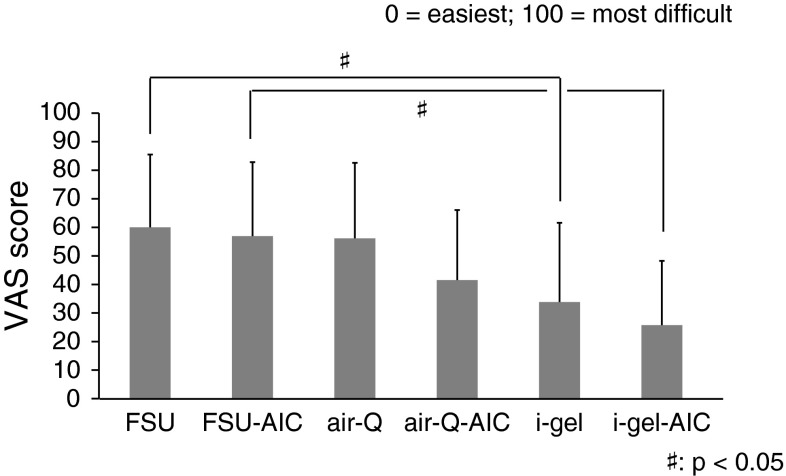
Difficulty of passing through the glottis (*n* = 21)

Figure 5 shows VAS scores for difficulty of passing through the glottis. There were no significant differences in scores with or without AIC use for any of the ISGAs. However, as with the difficulty of the entire intubation procedure already discussed, the FSU had a significantly higher VAS score for passing though the glottis compared with the i-gel (*p* < 0.05).

## Discussion

In the ASA Difficult Airway Algorithm 2003, the LMA was listed as an important alternative device for ventilation during the management of difficult airways. Since then, numerous SGAs have been introduced and included as must-have items in the 2013 ASA algorithm [12]. The findings of the present study suggest that ISGAs are advantageous in the management of difficult intubation cases. First, the airway can be secured with the SGA, followed by ventilation, which allows for intubation without the risk of hypoventilation and hypoxemia. Both clinical reports [18–20] and manikin simulation studies [23, 24] have been published on the use of SGAs with the AIC.

These previous reports demonstrate the utility of the AIC as a tube-exchanger for performing endotracheal intubation with an SGA. However, there have been no adequate studies on the effectiveness of the AIC as a fiberoptic guide of endotracheal tubes when used in combination with ISGAs. Furthermore, it remains unclear which ISGA is the most effective and whether simultaneous AIC use is a valid approach.

Against this backdrop, we examined the utility of the AIC with three different ISGAs used in clinical anesthesia, using a manikin. Given that most, if not all, of those who perform fiberoptic intubation have basic clinical experience with anesthesia, we limited our participants to medical doctors with more than 2 years of experience in clinical anesthesia.

The intubation times in FSU groups are significantly longer than those in air-Q and i-gel groups with or without AIC use. The difference between an FSU and another ISGA for mean intubation time might be dependent on the curved conduit of FSU. Concerning the role of AIC, we found no significant difference in intubation time and success rate with each ISGA with or without AIC use. However, we found that the cases in conjunction with the AIC significantly decreased the number of collisions with the glottis in both groups of the FSU and the air-Q. With respect to the i-gel, it may be suited for passing the tracheal tube through the manikin’s airway. The difficulty evaluation of passing through the glottis by the participants seems to have some relationship to the intubation time and the number of collisions of the tracheal tube with the glottis, which might have influenced the difficulty of the whole procedure because there is the same significant difference between the FSU groups and the i-gel groups. However, there is no significant difference with or without using the AIC, which result may be affected by several factors: we suppose that such are the simple technique without AIC and no significant difference of time to perform intubation regardless of AIC in each ISGA, besides the number of collisions with the glottis. When we consider clinical cases, it is very important to minimize the damage of the glottis in addition to shortening the intubation procedure. The possibility of vocal cord injury by collision of the tube tip with the glottis during intubation, and consequently the chance of developing laryngeal edema and subluxation of arytenoid cartilage consequent to mechanical friction, are expected to be reduced.

One limitation of this study was that 8 % lidocaine may degrade a fiberscope; we have to consider more about lubricants when we use AIC. Another limitation was that intubation was performed on a manikin. The manikin presents a nondifficult airway model in which intubation can be performed without retroflexion of the neck. Future studies with clinical cases are warranted.

## Conclusion

In conclusion, concerning these three SGAs, the cases in which FSU was used showed the longest times and the maximum number of collisions with the glottis to perform fiberoptic tracheal intubation. The findings of this study suggest that the AIC has clinical significance in that it decreases collisions of the tracheal tube with the glottis.
